# Baseline presentation of atypical anorexia nervosa in Singaporean adolescents: a retrospective cohort study

**DOI:** 10.1186/s40337-023-00943-4

**Published:** 2023-12-08

**Authors:** Chu Shan Elaine Chew, E. Eric Tay, Mei En Hannah Marian Lie, Khairunisa Binte Khaider, Courtney Davis

**Affiliations:** 1 Adolescent Medicine Service, KK Women’s and Children’s Hospital, 100 Bukit Timah Road, Singapore, 229899 Singapore; 2 SingHealth Duke-NUS Paediatric Academic Clinical Programme, Singapore, Singapore; 3 Duke NUS Medical School, Singapore, Singapore; 4 Lee Kong Chian School of Medicine, Singapore, Singapore

**Keywords:** Atypical anorexia nervosa, Anorexia nervosa, Adolescents, Eating disorder, Adolescents

## Abstract

**Background:**

While atypical anorexia nervosa (AAN) has been found to present with significant physical and psychological complications, the presentation of AAN has not been described in a multi-ethnic Singaporean population.

**Methods:**

This retrospective cohort analysis aimed to characterize the baseline presentation of adolescents with anorexia nervosa (AN) (N = 317) and AAN (N = 141) in a Singaporean cohort that presented to a specialist paediatric eating disorder program between January 2010 and October 2020 for assessment.

**Results:**

In patients with AAN, there were increased proportions of males (16% vs. 7%) and of Malay ethnicity (11% vs. 4%) compared to AN. Compared to adolescents with AN, adolescents with AAN had lower rates of admission (61% vs. 81%), bradycardia (45% vs. 75%), and hypotension (7% vs. 21%) but had a higher rate of syncope (13% vs. 7%). Likewise, adolescents with AAN had higher rates of self-harm and drug overdose (14% vs. 1.5%) requiring admission, more purging (45.1% vs. 14.8%) and more shape concerns.

**Conclusion:**

Highlighting the severity of the illness, Singaporean adolescents with AAN presented with physical complications of malnutrition and had more severe eating disorder psychopathology and a higher frequency of other psychological comorbidities than did adolescents with AN.

## Abstract (plain language summary)

Singaporean adolescents with atypical anorexia nervosa experience both physical and psychological complications of the illness.

## Introduction

Literature has described an increasing incidence of adolescents with atypical anorexia nervosa (AAN) in recent years [1–3]. Based on DSM-5 criteria, individuals with AAN meet the criteria for Anorexia Nervosa (AN), with the exception that their presenting Body Mass Index (BMI) lies within or above the average range despite significant weight loss [4, 5]. Previously classified as Eating Disorder Not Otherwise Specified (EDNOS) in DSM-IV, AAN has been classified as a subtype of Other Specified Feeding and Eating Disorder (OSFED) in DSM-5 [4]. An Australian study in 2010 documented more than a five-fold increase (from 8 to 47%) in proportion of patients with AAN among hospitalized adolescents over a 6 years period [3]. Although most research on AAN has been conducted in samples composed predominantly of Caucasian females [2, 6–13], literature suggests that the prevalence of AAN may be higher in males [6–11, 14] and non-Caucasian populations [1, 6, 7, 12–14].

While it is well established that individuals with AN are at risk for significant medical complications due to malnutrition and low body weight, there is growing recognition that significant or rapid weight loss can lead to similar complications for individuals with AAN even though the latter are in a normal or above-normal weight range [3]. Individuals with AAN experience similar rates of medical instability from malnutrition (such as bradycardia and hypotension) [2–15] and lower rates of menstrual disturbances than individuals with AN [2, 6, 14, 16, 17]. Moreover, eating-specific psychopathology in individuals with AAN is reported to be comparable to, or even higher than, that in individuals with AN, whereas the two groups show comparable levels of generalized psychopathology [3, 8, 11, 16, 18–20].

Although literature has indicated that individuals with AAN are at risk for medical instability [3, 16], few studies have examined the weight variables associated with medical instability in adolescents with AAN and AN. In predominantly Caucasian samples of adolescents with AN and AAN, Whitelaw et al. [3] identified total and recent weight loss as predictors of bradycardia independent of presentation weight while Garber et al. [14] showed the rate of weight loss was associated with lower heart rate independent of admission weight.

While research suggests the demographic characteristics associated with AAN may differ from those seen in AN [1], little is known about the epidemiology, presentation, and predictors of illness severity of AAN in Asian populations [21]. Our study thus aims to describe the baseline characteristics and initial presentation of adolescents with AN and AAN in a multi-ethnic Singaporean population and to understand the factors associated with physical and psychological markers of illness severity in this sample.

## Methods and materials

### Subject selection

This retrospective chart audit examined the case notes of all patients below the age of 18, who presented for evaluation for an eating disorder in both the inpatient or outpatient settings at KK Women’s and Children’s Hospital between January 2010 and October 2020. Patients with Bulimia Nervosa, Avoidant/Restrictive Food Intake Disorder and other unspecified conditions were excluded from the review. Data for this study was used in accordance with guidelines from SingHealth Institutional Review Board.

### Eating disorder diagnoses

At the time of presentation, all patients participated in a multidisciplinary intake evaluation led by an adolescent medicine physician with eating disorder expertise. Prior to 2014, DSM IV criteria were used at the time of assessment to assign eating disorder diagnoses [22]. From 2014 onwards, DSM-5 criteria were used to assign eating disorder diagnoses.

At the time of the case audit, case notes and diagnoses were reviewed by an adolescent medicine physician and a psychologist with expertise in EDs to retrospectively assign a diagnosis using DSM-5 criteria for patients who were initially assessed using DSM-IV criteria [4] and to ensure the application of consistent BMI and weight loss criteria across all cases.

Low weight for AN was defined as a percent median Body Mass Index (mBMI%) of less than 87%. The preceding criterion has been applied in other studies on adolescent ED [23–25]. Patients who met all criteria for AN, but who had normal or above-normal BMI at presentation (defined as %mBMI of greater than 87%) despite significant weight loss were classified as AAN. Published criteria for severe malnutrition were used to establish the definition of significant weight loss [26].

### Patient variables at presentation

Ethnicity (Chinese, Malay, Indian, and Others including Caucasian and Filipino), highest historical weight and corresponding height, menstrual status, duration of weight loss, presence of purging, and use of weight loss medications were self-reported by patients and parents at first presentation. Measurements for height, weight, and an orthostatic measurement of heart rate and blood pressure (lying and standing) were abstracted from the clinical record. Duration of admission and reasons for it were recorded.

### Weight variables and menstrual history

Percent median BMI (%mBMI) was calculated using the BMI of the patient divided by the 50^th^ percentile BMI corrected for age and gender, and multiplied by 100. Fiftieth percentile BMI (%mBMI) for exact age was determined using the BMI-for-age growth charts for Singaporean children and adolescents aged 4 to 18 years [27]. Weight loss was defined as the change in %mBMI from highest %mBMI to the %mBMI at presentation. Duration of weight loss (months) was defined as the time interval between the date of highest weight and the presentation date. Rate of weight loss was the change in %mBMI divided by the duration of weight loss [14]. Secondary amenorrhea was defined as the disruption of previously regular menstruation cycles for 3 months or more [28].

### Admission criteria

Criteria for admission included physiologic instability such as bradycardia and hypotension as well as acute medical complications of malnutrition including syncope as per published international guidelines [29]. Syncope is defined by transient loss of consciousness and postural tone with spontaneous recovery [30]. Admission criteria also included the presence of significant safety concerns such as attempted self-harm or suicide requiring medical or safety monitoring. Patients admitted for medical instability were placed on ED care protocols for medical stabilization [24].

### Psychological measures

The Eating Disorder Examination Questionnaire (EDE-Q) [31] is a self-administered questionnaire with scores ranging from 1 to 9. Higher scores indicate greater severity of ED psychopathology. In our service, routine administration of the EDE-Q started in 2016 as part of the multidisciplinary assessment.. Psychologists or psychiatrists from the ED team evaluated for psychiatric comorbidities including symptoms of anxiety and depression, suicidal ideation, and self-harm. Only diagnoses assigned by the treating psychologist or psychiatrist were abstracted from the case notes.

### Statistical analysis

Data was analysed using SPSS software version 19 for Windows (SPSS Inc., Chicago, IL). Baseline demographic and anthropometric measurements of study participants were compared between patients with AN and AAN using two independent samples *t* tests (or Wilcoxon rank sum, depending on normality) and chi-square tests (or Fisher exact test, where appropriate) for continuous and categorical variables respectively. Data were presented as mean ± standard deviation for continuous variables, and frequencies and percentages for categorical variables. The number of adolescents with AAN was presented as a proportion of the total AN and AAN cases along with a line of best fit. Associations between four weight history variables (independent variables) and markers of illness severity were examined with multiple regression models. The four independent weight variables were: (1) %mBMI (2) Change in %mBMI (3) Rate of weight loss (4) Duration of weight loss. Models were adjusted for gender, age and race. Multivariable linear regression, followed by stepwise backward selection, was used to examine the contribution of the weight variables on illness severity outcomes measures. Measures of illness severity were vital signs at presentation (heart rate and systolic blood pressure), duration of admission, and ED psychopathology (global EDE-Q scores).

## Results

**Fig. 1:**
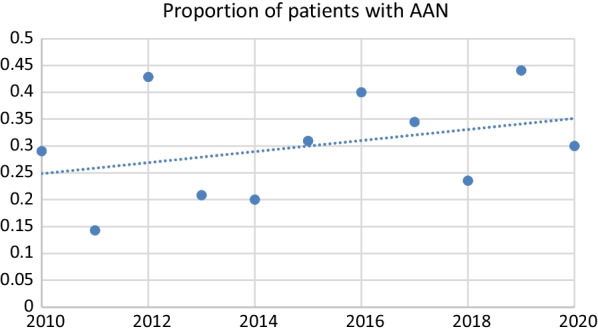
Scattergraph plot showing proportion of AAN relative to total cases of AAN and AN per year with a line of best fit

The study population consisted of 459 adolescents with a mean age of 14.1 (± 1.5) years (range 9–18 years) and a mean duration of illness of 8.1(± 6.2) months. The study sample was 90.2% female with 318 patients (62.7%) diagnosed with AN and 141 patients (27.8%) diagnosed with AAN. There was a greater proportion of males (15.6% vs. 7.2%, *p* = 0.007) and individuals of Malay ethnicity (10.6% vs. 3.5%, *p* = 0.025) diagnosed with AAN compared to AN. Table 1 details the baseline characteristics for patients with AN and AAN and admission information. Between 2010 and 2020, an increasing proportion of patients was diagnosed with AAN relative to the total number of cases of AN and AAN per year. Figure 1 shows the proportion of patients diagnosed with AAN relative to the total number of cases of AN and AAN for each year of the study period.

**Table 1:** Baseline characteristics of AN and AAN at presentation

	AN n = 318 (62.7%)	AAN n = 141 (27.8%)	OR or mean difference (95% CI)	*p* value
	N (%) or Mean ± SD	N (%) or Mean ± SD		
Females	295 (92.8%)	119 (84.4%)	2.371 (1.273 to 4.417)	0.007*
*Ethnicity*				
Chinese	260 (81.8%)	97 (68.8%)	Reference	
Malay	11 (3.5%)	15 (10.6%)	3.655 (1.622 to 8.235)	0.025*
Indian	25 (7.9%)	15 (10.6%)	1.608 (0.814 to 3.179)	0.717
Others	22 (6.9%)	14 (9.9%)	1.706 (0.839 to 3.468)	0.883
Age, years	14.08 ± 1.56	14.06 ± 1.332	0.018 (− 0.279 to 0.315)	0.906
*Weight variables*				
Premorbid overweight	16 (5.5%)	39 (32.2%)	8.234 (4.377 to 15.490)	< 0.001*
BMI at presentation	14.46 ± 1.47	18.84 ± 2.19	− 4.382 (− 4.727 to − 4.037)	< 0.001*
%mBMI at presentation	74.60 ± 7.27	97.10 ± 9.95	− 22.566 (− 24.199 to − 20.932)	< 0.001*
Weight loss (Change in %mBMI)	21.59 ± 12.26	21.04 ± 15.87	0.550 (− 2.30 to 3.40)	0.705
Duration of weight loss (months)	7.98 ± 6.01	8.27 ± 6.49	− 0.294 (− 1.62 to 1.03)	0.539
Rate of weight loss, (change in %mBMI/month)	3.76 ± 2.94	3.56 ± 3.19	0.206 (− 0.45 to 0.87)	0.663
*Menstrual history*				
Secondary amenorrhea amongst females	168 (57%)	47 (39.5%)	0.161 (0.072 to 0.357)	< 0.001*
*Admission information*				
Requires admission	259 (81.4%)	86 (61%)	2.807 (1.806 to 4.363)	< 0.001*
Duration of admission, days	12.12 ± 6.59	8.91 ± 5.80	3.213 (1.645 to 4.781)	< 0.001*
*Reasons for admission*				
Medical instability				
Bradycardia	195(75.3%)	39 (45.4%)	0.272 (0.164 to 0.453)	< 0.001*
Hypotension	55 (21.2%)	6 (7%)	0.278 (0.115 to 0.672)	0.004*
Orthostatic hypotension	7(2.7%)	0(0%)	NA	0.125
Syncope	21 (6.6%)	18 (12.8%)	2.070 (1.066 to 4.020)	0.032*
*Psychological safety concerns*				
Self-harm/Suicide attempt/Drug overdose	4 (1.5%)	12 (14%)	10.338 (3.238 to 33.003)	< 0.001*

*%mBMI* percent median body mass index, *NA* Not applicable

^*^p < 0.05

### Weight variables and menstrual status

There was no significant difference in the duration, amount, or rate of weight loss between adolescents with AN or AAN. Adolescents with AAN were more likely to be premorbidly overweight (32.2% vs. 5.5%, *p* < 0.001). Adolescents with AAN were also less likely to present with secondary amenorrhea (57% vs. 39.5%, *p* =  < 0.001). Please see Table 1 for the baseline characteristics of adolecsents with AN and AAN at first presentation.

### Admission rate and reasons for admission

Adolescents with AAN were less likely to be admitted at first presentation compared to adolescents with AN (81.4% vs. 61%, *p* < 0.001). Adolescents with AAN were more likely to be admitted for self-harm or suicide ideation (14% vs. 1.5%, *p* < 0.001), and less likely to be admitted for bradycardia (21.2% vs. 7%, *p* = 0.004) or hypotension (7% vs. 0%, *p* = 0.125). Adolescents with AAN had higher rates of syncope (12.8% vs. 6.6%, *p* = 0.032) compared to adolescents with AN. Adolescents with AAN also had shorter durations of inpatient stay (AN: 12.12 ± 6.59 days; AAN: 8.91 ± 5.80 days, *p* < 0.001). Table 1 outlines the reasons for admission in adolescents with AN and AAN.

### Psychological measures

Table 2 outlines the baseline psychological characteristics for adolescents with AAN and AN. Compared to adolescents with AN, a significantly higher proportion of adolescents with AAN presented with depression, deliberate self-harm and suicidal ideation (*p* < 0.05 for all analyses). Purging was more common among adolescents with AAN (45.1% vs. 14.8%, *p* < 0.001). Adolescents with AAN showed trends towards worse EDE-Q scores than did adolescents with AN in all domains, with EDE-Q shape concerns being significantly higher in AAN cases (AN: 3.2 ± 2.1; AAN: 4.1 ± 1.7; *p* = 0.043).

**Table 2:** Psychological features of AN and AAN at baseline presentation

	AN n = 318 (62.7%)	AAN n = 141 (27.8%)	OR or mean difference (95% CI)	*p* value
	N (%) or Mean ± SD	N(%) or Mean ± SD		
*Psychological presentation*				
Anxiety symptoms	61 (19.2%)	25 (17.7%)	0.908 (0.543 to 1.519)	0.713
Depressive symptoms	37 (11.6%)	34 (24.1%)	2.413 (1.440 to 4.044)	0.001
Deliberate self-harm	29 (9.1%)	24 (17%)	2.037 (1.138 to 3.646)	0.017
Active/Passive suicide ideation	20 (6.3%)	18 (12.8%)	2.173 (1.111 to 4.249)	0.023
Purging	13 (14.8%)	23 (45.1%)	4.739 (2.115 to 10.619)	< 0.001
Use of weight loss medications	13 (5.7%)	8 (7.4%)	1.329 (0.534 to 3.309)	0.541

Eating disorder examination questionnaire	Mean ± SD	Mean ± SD	Mean difference Δ (95% CI)	
	AN n = 46	AAN n = 30		
Restraint	2.5 ± 1.77	2.9 ± 1.62	− 0.425 (− 1.230 to 0.380)	0.288
Eating concern	2.1 ± 1.40	2.7 ± 1.60	− 0.568 (− 1.260 to 0.126)	0.118
Shape concern	3.2 ± 2.07	4.1 ± 1.73	− 0.904 (− 1.813 to 0.004)	0.043*
Weight concern	2.9 ± 1.90	3.6 ± 1.84	− 0.714 (− 1.590 to 0.163)	0.107
Global Score	2.7 ± 1.60	3.4 ± 1.63	− 0.735 (− 1.490 to 0.020)	0.058

^*^p < 0.05

### Associations between weight variables and markers of illness severity

Lower presenting %mBMI was associated with lower heart rate (β = 0.248 [CI 0.047 to 0.449]; *P* = 0.016), lower systolic blood pressure (β = 0.610 [CI 0.302 to 0.918]; *p* ≤ 0.001), higher (worse) EDE-Q shape concern scores (β = − 5.231 [CI − 10.013 to − 0.450]; *p* = 0.043) and longer duration of admission (β = − 1.033 [CI − 1.646 to − 0.419]; *p* = 0.001). There were no significant associations between markers of illness severity and amount of weight loss, rate of weight loss, or duration of weight loss (Table 3).

**Table 3:** Associations between presenting %mBMI, weight loss, rate of weight loss and duration of weight loss with markers of illness severity

Markers of illness severity	%mBMI		Weight loss		Rate of weight loss		Duration of weight loss	
	Unadjusted	Adjusted^a^	Unadjusted	Adjusted^a^	Unadjusted	Adjusted^a^	Unadjusted	Adjusted^a^
	β (CI)	β (CI)	β (CI)	β (CI)	β (CI)	β (CI)	β (CI)	β (CI)
*Presenting vital signs*								
Heart rate	0.228* (0.033 to 0.423)	0.215* (0.016 to 0.414)	0.060 (− 0.180 to 0.300)	0.082 (− 0.147 to 0.311)	− 0.014 (− 0.062 to 0.034)	− 0.022 (− 0.070 to 0.026)	0.033 (− 0.043 to 0.108)	0.024 (− 0.056 to 0.104)
Systolic BP	0.389* (0.098 to 0.680)	0.448* (0.156 to 0.740)	0.200 (− 0.151 to 0.552)	0.137 (− 0.203 to 0.478)	0.040 (− 0.032 to 0.111)	0.063 (− 0.007 to 0.133)	− 0.045 (− 0.158 to 0.067)	− 0.057 (− 0.174 to 0.060)
Duration of admission	− 1.110** (− 1.715 to− 0.505)	− 1.439** (− 2.097 to− 0.780)	− 0.279 (− 1.071 to 0.514)	− 0.057(− 0.767 to 0.652)	0.081 (− 0.067 to 0.230)	0.028 (− 0.130 to 0.186)	− 0.102(− 0.337 to 0.132)	− 0.046(− 0.053 to− 0.348)
EDE-Q global scores	2.257* (0.592 to 3.923)	2.335* (0.704 to 3.966)	− 0.386 (− 2.350 to 1.578)	− 0.463 (− 2.415 to 1.489)	− 0.011 (− 0.421 to 0.398)	0.012 (− 0.380 to 0.404)	0.108 (− 0.538 to 0.765)	0.111 (− 0.542 to 0.765)

^a^Multivariable model adjusted for age, gender and race

*P < 0.05

**P < 0.01

## Discussion

This is the first study in a multi-ethnic Asian population to characterize differences in physical and psychological characteristics on presentation between adolescents with AN and AAN. Compared to AN, we found a higher proportion of males and adolescents of Malay ethnicity with AAN. Despite presenting at a normal or high body weight, adolescents with AAN showed physical consequences of malnutrition and more psychological disturbances than adolescents with AN.

Consistent with other studies [2, 3, 14, 16], our study found patients with AAN presented with lower rates of both vital sign instability requiring hospital admission and secondary amenorrhea than their AN counterparts, despite having similar amount, duration, and rate of weight loss. However, this does not mean that adolescents with AAN do not suffer from complications of malnutrition as approximately half of our adolescent sample with AAN required admission for medical stabilisation. Interestingly, we also noted that adolescents with AAN were more likely to present with syncope compared to adolescents with AN. Given the increasing numbers of patients presenting with AAN, it is important for healthcare providers to screen for a history of weight loss and restrictive eating in adolescents with normal or higher body weight presenting with syncope or pre-syncopal changes.

Similar to the study by Garber et al. [14], we found that a lower %mBMI at presentation was associated with lower heart rate and higher EDE-Q scores. However, we did not find similar associations with the amount, duration or rate of weight loss [3, 14]. The lack of association of markers of illness severity with the amount, duration and rate of weight loss in our study may be explained by differences in the sample populations; our study looked at a wider spectrum of illness including both inpatient and outpatient presentations in the cohort.

Our study found that Asian adolescents with AAN experienced higher rates of depression, self-harm and suicidal ideation at presentation than did those with AN, with a greater proportion requiring admission for psychological safety reasons. This is in contrast to the findings of recent research that did not find an increase in non-ED related psychopathology amongst patients with AAN compared to patients with AN [2, 16]. In our setting, we have observed adolescents with AAN more commonly present to care for evaluation of their mood issues, self-harm and suicidal ideations rather than for evaluation of their weight loss. This may contribute to the increased proportion of non-ED psychopathology among adolescents with AAN in our study.

Consistent with findings from other studies, we noted trends towards worse EDE-Q scores on all domains in adolescents with AAN versus AN, with significantly higher scores in the Shape Concern domain amongst adolescents with AAN [2, 14, 16]. Of concern, our study also found more Asian adolescents with AAN engaging in purging behavior compared to adolescents with AN. The preceding contrasts with findings of the study by Sawyer et al. [16] which found similar rates of purging amongst participants with AAN and AN.

Our study found a higher percentage of males and those of Malay ethnicity among the AAN subgroup versus among the AN subgroup. This observation appears to mirror the high rates of obesity among males and among those of Malay ethnicity in Singapore [32]. Many shared risk factors have been identified between obesity and EDs, including body dissatisfaction, dieting, weight control behaviours and low self-esteem. Studies have shown that adolescents with adverse childhood experiences, which include bullying and body-related difficulties, have higher odds of developing AAN [10]. The potential association between overweight and eating disorders reinforces the need for a holistic and non-stigmatising approach to adolescent obesity programs and public health prevention programs.

While we did not find a dramatic increase in the proportion of adolescents presenting with AAN compared to AN, we believe this reflects a gap in help-seeking among adolescents with AAN in Singapore. Notably, a recent cross-sectional prevalence study found a high prevalence of OSFED (37%) and low rates of help-seeking (1.6%) amongst Singaporean adults [33]. In our experience, adolescents with AN commonly present to care as a result of their low body weight. In these cases, families, school staff, and friends encourage the affected adolescent to seek help for their weight loss. In contrast, many adolescents with AAN are complimented by families, school staff, and friends for weight loss and are often encouraged to continue their weight loss. This general lack of public awareness of AAN likely contributes to the lower rates of help seeking behavior and further highlights the importance of prevention and early intervention programs for ED.

## Limitations and strengths

The retrospective nature of this study presents several inherent limitations. Data recorded in the electronic medical record were not always complete and depended on the original clinical documentation. Eating disorder diagnoses were assigned by a multidisciplinary team as part of routine clinical care and retrospectively adjusted to DSM-5 diagnoses by the authors as required. This may impact the reliability of the diagnoses and the generalizability of the findings. As routine administration of the EDE-Q started in 2016, the available sample size of patients with EDE-Q data is limited which may also affect the generalizability of the results obtained. Finally, psychiatric diagnoses only included those that were labelled in the medical records by the team psychologists and psychiatrists and subclinical psychiatric diagnoses were not included in the data collection. This may lead to an under-representation of the burden of non-ED psychopathology.

Strengths of our study included this being the first study to investigate the differences in presentation of a large cohort of a multi-ethnic population of Singaporean adolescents with AAN and AN and the inclusion of inpatient and outpatient cases.

## Conclusion

This study highlights how AN and AAN present differently in Singaporeans with males and Malay ethnicity presenting more commonly with AAN than AN. Adolescents with AAN present less frequently with features of medical instability requiring admission compared to adolescents with AN. However, adolescents with AAN present more frequently with psychological safety concerns requiring admission in our study. We also found higher rates of depression and self-harm with worse eating disorder psychopathology and more common purging behaviours in adolescents with AAN. These findings are instructive in guiding the identification and management of Asian adolescents with suspected AAN.

## Data Availability

The datasets used and analysed during the current study are available from the corresponding author on reasonable request.

## Abbreviations

AAN: Atypical anorexia nervosa
AN: Anorexia nervosa
EDE-Q: Eating disorder examination questionnaire
DSM-5: Diagnostic and statistical manual of mental disorders, 5th edition
%mBMI: Percent median body mass index
ED: Eating disorder
EDNOS: Eating disorder not otherwise specified
